# Supplementary material for: On the correspondence between the transcriptomic response of a compound and its effects on its targets
Source: BMC Bioinformatics. 2023 May 19;24:207. doi: 10.1186/s12859-023-05337-6 (PMC10199609; doi:10.1186/s12859-023-05337-6)
Supplement: Supplementary file 2 — Additional file 2. Supplementary text and figures. [file 12859_2023_5337_MOESM2_ESM.pdf]

Supplementary Figures

Algorithm 1 Adding pathway information to target vectors

```
Variables
▷ targets_df is a data frame where each row corresponds to the target vector for a specific compound
▷ pathway_df is a data frame mapping each protein to other protein(s) present in the same gene sets (pathways). Pathways/Gene sets are taken from the KEGG, WikiPathways, and Reactome databases.

procedure ADD_PATHWAYS(targets_df, pathways_df)
    new_targets_df ← copy(targets_df)
    for each compound do
        for each target of the compound do
            value ← targets_df[compound, target]
            pathway_proteins ← proteins in pathways containing the target
            for protein in pathway_proteins do
                if new_targets_df[compound, protein] = 0 then
                    new_targets_df[compound, protein] ← value
    return new_targets_df
```

Supplementary Figure 1. Pseudocode for enriching target vectors with pathway information from KEGG, WikiPathways and Reactome.

Algorithm 2 Adding topological pathway information to target vectors

```
Variables
▷ targets_df is a data frame where each row corresponds to the target vector for a specific compound
▷ topological_pathway_df is a data frame containing topology of a protein-protein interaction network. Each row contains a source protein, a targeted protein, and their relation (up-regulated or down-regulated)

procedure ADD_PATHWAYS(targets_df, topological_pathway_df)
    n ← number of times to repeat (Supplementary Figure 3)
    new_targets_df ← copy(targets_df)
    for i = 0, . . . , n do
        for each compound do
            target_vector ← new_targets_df[compound]
            all_targets ← indexes of non-zero entries (targets) in target_vector
            for t in all_targets do
                value ← targets_df[compound, t]
                df ← rows of topological_pathway_df where t is the source
                num_rows ← number of rows in df
                for j = 0, . . . , num_rows do
                    protein ← df[j, targeted]
                    if new_targets_df[compound, protein] = 0 then
                        new_targets_df[compound, protein] ← value*df[j, relation]
    return new_targets_df
```

Supplementary Figure 2. Pseudocode for adding information from protein-protein interaction networks to the target vectors.

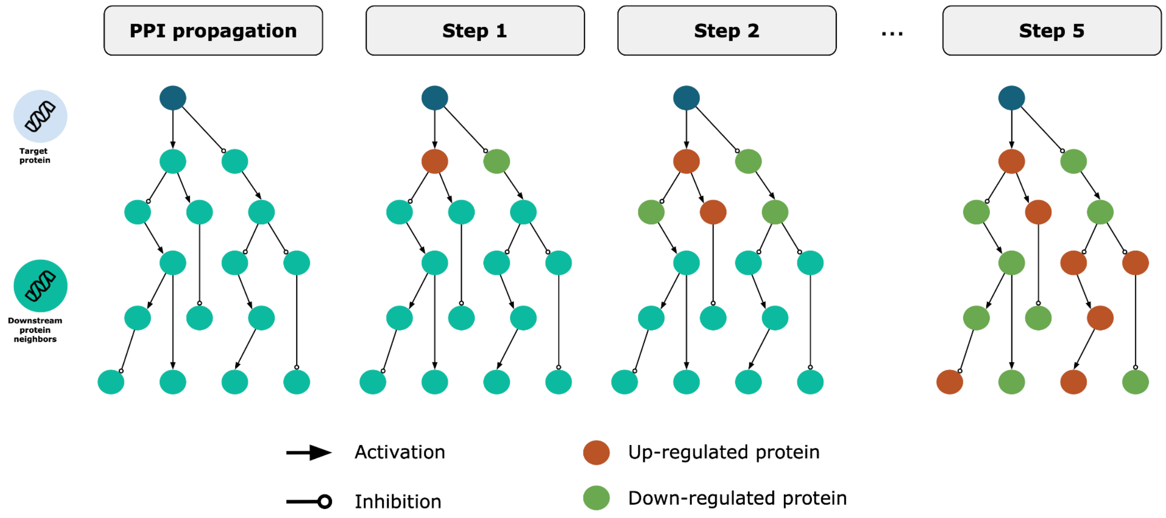

Supplementary Figure 3. Propagation of the activatory/inhibitory effect through protein-protein interactions derived from KEGG.

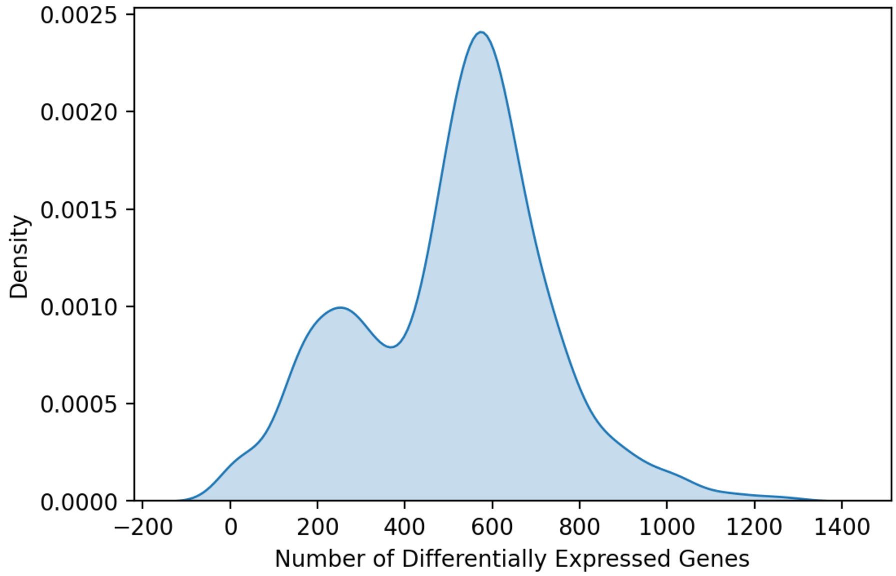

Supplementary Figure 4. Distribution of differentially expressed genes for each chemical

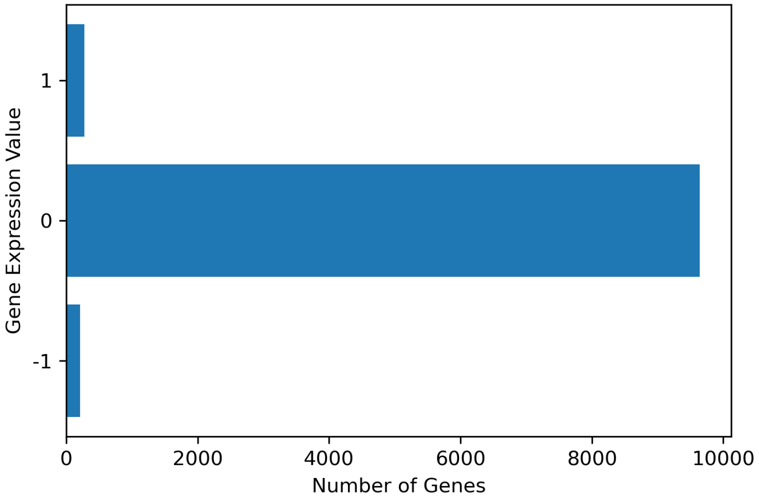

Supplementary Figure 5. Gene expression values for known targets in the ChemPert database.

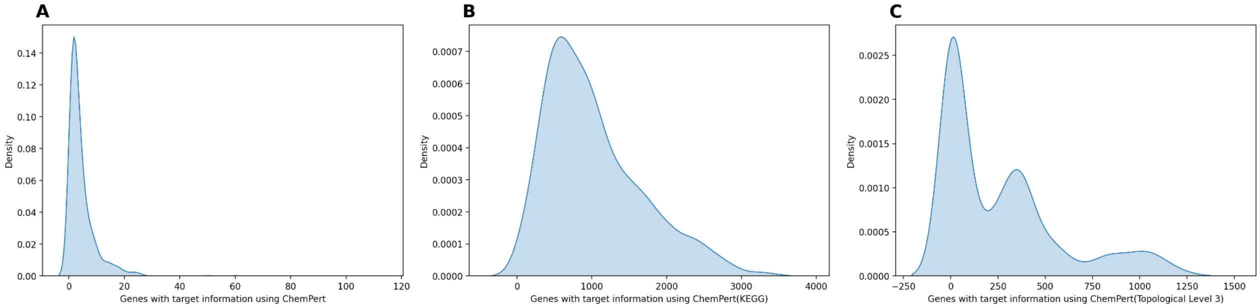

Supplementary Figure 6. a) Distribution of targets for each chemical using the original data from the ChemPert database. b) Distribution of targets for each chemical after enriching them with the gene sets from the KEGG database. c) Distribution of targets for each chemical after the enrichment using topological information up to three levels downstream of the target(s).

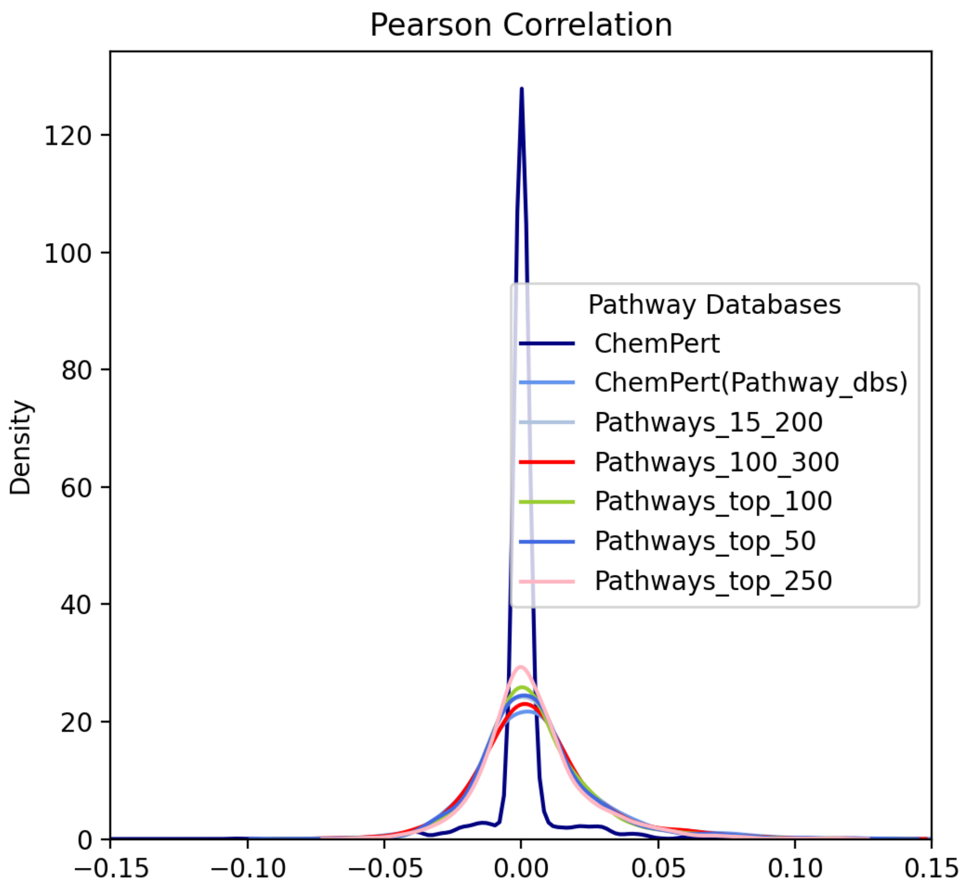

Supplementary Figure 7. Pearson correlation scores for pathway and transcriptomic vectors from ChemPert for each of the 2,512 compounds using different subsets of pathways. We first tried keeping pathways from the dataset with 100-300 genes (Pathways_100_300). When we did this, we were only left with 300 pathways (1660 pathways are kept when we use pathways with 15-300 genes). Additionally, we tried keeping pathways with 15-200 genes. This resulted in a set of 1600 pathways. We also tried removing the pathways with the top 50,100, and 250 number of genes (Pathways_top_X where X is the number of pathways we removed). We used each of these sets of pathways to adjust the target vectors (as described in section 2.2.2) and computed the Pearson correlation and Jaccard similarity between these new target vectors and the transcriptomic response vectors.

Supplementary Text

The ChemPert database includes 82,270 transcriptional signatures from 167 different cell types. The data we used was generated from 2,508 unique perturbagens. They collected data about the targets of different perturbagens from Drugbank, STITCH, and Drug repurposing Hub. The perturbagens that were used included both chemical and biological perturbagens. Information about the gene expression of different cell types and the transcriptional responses was collected from Gene Expression Omnibus (GEO), ArrayExpress, and LINC L1000. The dataset of transcriptional responses was manually curated and included responses from non-cancerous cells in humans, mice, and rats. For more information about how the data was collected and processed see the original work (Zheng *et al.*, 2022).
